# Alterations in ocular microcirculation and oxygen metabolism in patients with lipemia retinalis

**DOI:** 10.1186/s12886-022-02515-7

**Published:** 2022-07-06

**Authors:** Waleed K. Alsarhani, Fadwa F. Al Adel, Abdullah Alamri, Rahaf M. Al Malawi, Abdulrahman F. AlBloushi

**Affiliations:** 1 Department of Ophthalmology and Vision Sciences, University of Toronto, Toronto, ON Canada; 2 Department of Ophthalmology, King Faisal Specialist Hospital & Research Centre, Riyadh, Saudi Arabia; 3 Department of Ophthalmology, College of Medicine, Princess Nourah Bint Abdulrahman University, Riyadh, Saudi Arabia; 4 Department of Ophthalmology, King Abdullah bin Abdulaziz University Hospital, Riyadh, Saudi Arabia; 5 College of Computer and Information Sciences, Princess Nourah Bint Abdulrahman University, Riyadh, Saudi Arabia; 6 Department of Ophthalmology, College of Medicine, King Saud University, Riyadh, Saudi Arabia

**Keywords:** Lipemia retinalis, Laser speckle flowgraphy, Retinal oximetry

## Abstract

**Purpose:**

The study aims to assess the alterations in retinal oxygen saturation and retinal and choroidal blood flow in lipemia retinalis.

**Methods:**

This was a cross-sectional study on 10 eyes (5 patients) with history of lipemia retinalis. The study comprised 10 eyes with documented history of lipemia retinalis and 10 participants as healthy controls. Patients with a confirmed history of lipemia retinalis were grouped into two cohorts based on their most recent fundus examination: *untreated* lipemia retinalis (abnormal fundus) and *resolved* lipemia retinalis (normal fundus). Both retinal arteriolar and venular oxygen saturation were measured using the non-invasive spectrophotometric retinal oximeter (Oxymap T1). The mean blur rate (MBR) of the optic nerve and choroidal blood flow were analyzed using a laser speckle flowgraph (LSFG).

**Results:**

Patients with untreated lipemia retinalis had a significantly higher retinal arteriolar and venular oxygen saturation than that of the other two groups (*p* < 0.001). Moreover, patients with untreated lipemia retinalis had significantly smaller retinal arteriolar and venular diameters (*p* < 0.001). On LSFG, there was a significant difference in the overall MBR (*p* = 0.007) and vessel MBR of the optic nerve between the groups (*p* = 0.011). The patients with history of lipemia retinalis (untreated and resolved) exhibited a high overall MBR and vessel MBR of the optic nerve than that of the control group. There was a significant elevation of the optic nerve (*p* = 0.002) and choroidal blowout score (*p* < 0.001), while the resistivity index of the optic nerve (*p* = 0.001) and choroids (*p* = 0.002) was significantly lower in patients with resolved and untreated lipemia retinalis.

**Conclusions:**

There was a significant alteration in retinal oximetry, in untreated lipemia retinalis, and in retinal blood flow, in both the resolved and untreated groups. The increase in retinal blood flow and oxygen saturation may elucidate the preservation of visual acuity and function despite the fundus changes observed in lipemia retinalis.

## Introduction

Lipemia retinalis leads to the retinal vessel creamy discoloration, secondary to hypertriglyceridemia, specifically chylomicronemia, familial or secondary to other causes. It is generally an asymptomatic condition, not commonly affecting visual acuity [1]. Moreover, it is associated with primary and secondary hypertriglyceridemia [2, 3], with the patients affected at risk of developing pancreatitis and coronary artery disease [4]. Lipemia retinalis has been observed in different age groups, including infants and children [5–12]. Initially, creamy discoloration of vessels appears in the peripheral fundus and then progresses towards the optic disc. Furthermore, with markedly high levels of triglycerides, retinal arteries and veins become indistinguishable along with a salmon-colored fundus [13].

There have been previous reports regarding color fundus photography, spectral-domain optical coherence tomography (SD-OCT), multicolor laser scanning, and electrophysiologic findings in lipemia retinalis [14–17]. However, to the best of our knowledge, analysis of the microcirculation and retinal oxygenation in lipemia retinalis has not been reported previously. Retinal oximetry that measures hemoglobin oxygen saturation of the retinal blood vessels and blood flow has been studied in retinal vascular diseases such as central retinal vein occlusion. Retinal hypoxia was observed in central retinal vein occlusion and correlated with decreased visual acuity. Although there is prominent retinal and vascular discoloration, visual acuity remains unaffected in lipemia retinalis. The study aimed to evaluate if retinal blood flow or oxygen saturation changes may elucidate the discrepancy between visual acuity and fundus appearance in lipemia retinalis.

## Methods

The study was conducted at King Saud University Medical City, Riyadh, Saudi Arabia. Retinal oxygenation was measured using the dual-wavelength (570 nm and 600 nm) non-invasive spectrophotometric retinal oximeter Oxymap T1 (Oxymap, EHF, Reykjavik, Iceland). The machine comprises a fundus camera (Topcon TRC-50 DX; Topcon, Tokyo, Japan), an image splitter, and two digital cameras in addition to analysis software (Oxymap Analyzer software, Oxymap EHF). Measurements were made between two circles within the optic disc generated by the software. The circles were 1.5 and 3 times the optic nerve size. Retinal vessel diameters are represented in pixels (1 pixel = 9 µm). Since variability increases with smaller vessels, the software eliminates any vessel with a diameter less than 6 pixels (56 µm).

Retinal blood flow measurement was conducted using Laser Speckle Flowgraphy (LSFG)-RetFlow (Nidek Co., LTD, Camagori, Aichi, Japan). The analysis was performed using a fundus camera with an infrared diode laser, image sensor, and ordinary charged coupled device camera (infrared and high-resolution digital cameras) (LMD-1000; Victor Company of Japan, Tokyo, Japan). The LSFG technique has been described previously [18]. The primary outcome of LSFG is the mean blur rate (MBR), the blurring speckle pattern formed by the reflected light of retinal red blood cells. MBR measures the ocular blood speed in arbitrary units. Both retinal and choroidal MBRs were measured. Choroidal MBRs can be calculated by measuring LSFG in an area lacking retinal vessels (fovea) [19]. Two circles were generated using the software; the first circle was set manually on the optic nerve for assessing the retinal vasculature, while the second circle was applied to the temporal side of the optic nerve head to analyze the choroidal vasculature. The position and size of the circles were saved into the software, ensuring consistent analysis for all study participants. Blowout score (BOS) is the blood volume maintained between heartbeats. Resistivity index (RI) is the ratio of the difference between the maximum and minimum MBR divided by the maximum MBR. The description of each parameter was previously described in detail [20, 21].

Before conducting LSFG and Oxymap T1, patients with a confirmed history of lipemia retinalis and primary hyperlipidemia were examined by two retina specialists. Based on the results of the recent fundus examination, patients were grouped into; 1) Untreated lipemia retinalis group (if the latest fundus examination was abnormal) or 2) Resolved lipemia retinalis group (if the latest fundus examination was normal). The patients in the latter group had hypertriglyceridemia along with a previous history of the abnormal fundus; however, using the lipid-lowering therapy, the fundus regained its normal appearance. The former group were just started on lipid lowering medications however their fundus appearance was still abnormal at the time of the study. SD-OCT (Heidelberg Engineering, Heidelberg, Germany) and swept-source OCT-angiography (Triton, Topcon, Tokyo, Japan) were conducted on both groups. Serum triglyceride levels were measured in miligram per deciliters for both the untreated and resolved groups. The study included six eyes (three patients) in the resolved group, four eyes in the untreated group (two patients), and twenty healthy control eyes (10 participants) were used for comparisons. Participants in the control group were interviewed to review their past medical history, and they were generally healthy. All control patients were examined to exclude the presence of any ocular disease before performing LSFG or retinal oximetry Oxymap T1.

Statistical analysis was performed using the Statistical Package for Social Science 27.0. The data were normally distributed, and analysis of variance with post- hoc analysis was used to compare the means of retinal oximetry and LSFG findings between different groups. A *p*-value < 0.05 was considered to be statistically significant. The study was conducted in adherence to tenets of the Declaration of Helsinki for research involving human participants after obtaining written informed consent from each patient.

## Results

### Untreated lipemia retinalis group

**Fig. 1:**
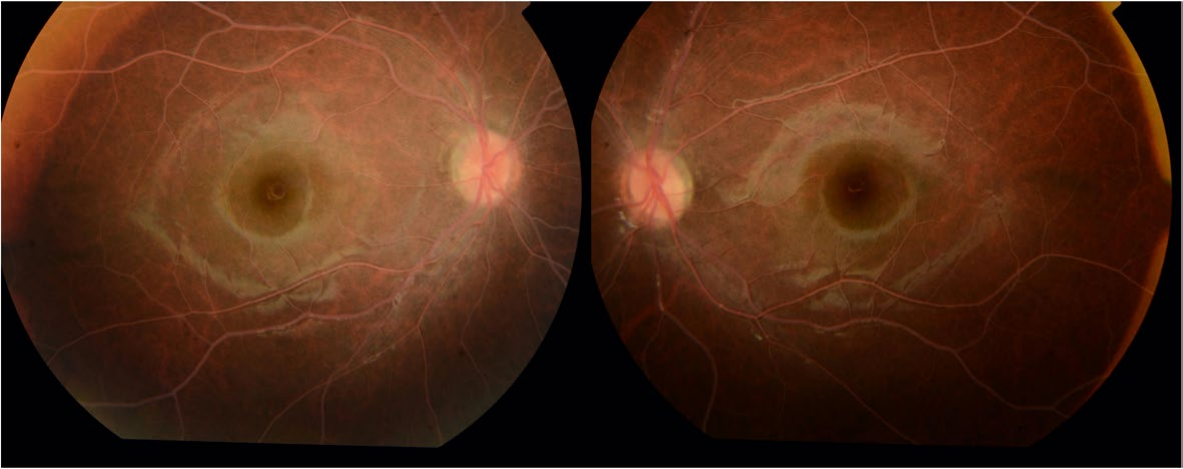
Color fundus photographs of bilateral eyes demonstrating a creamy discoloration of the blood vessels consistent with lipemia retinalis

**Fig. 2:**
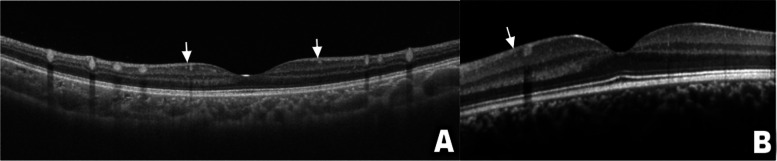
Spectral-domain optical coherence tomography revealing the presence of hyperreflective dots (arrows) in the inner retina in patients with untreated (**a**) and resolved lipemia retinalis (**b**)

**Fig. 3:**
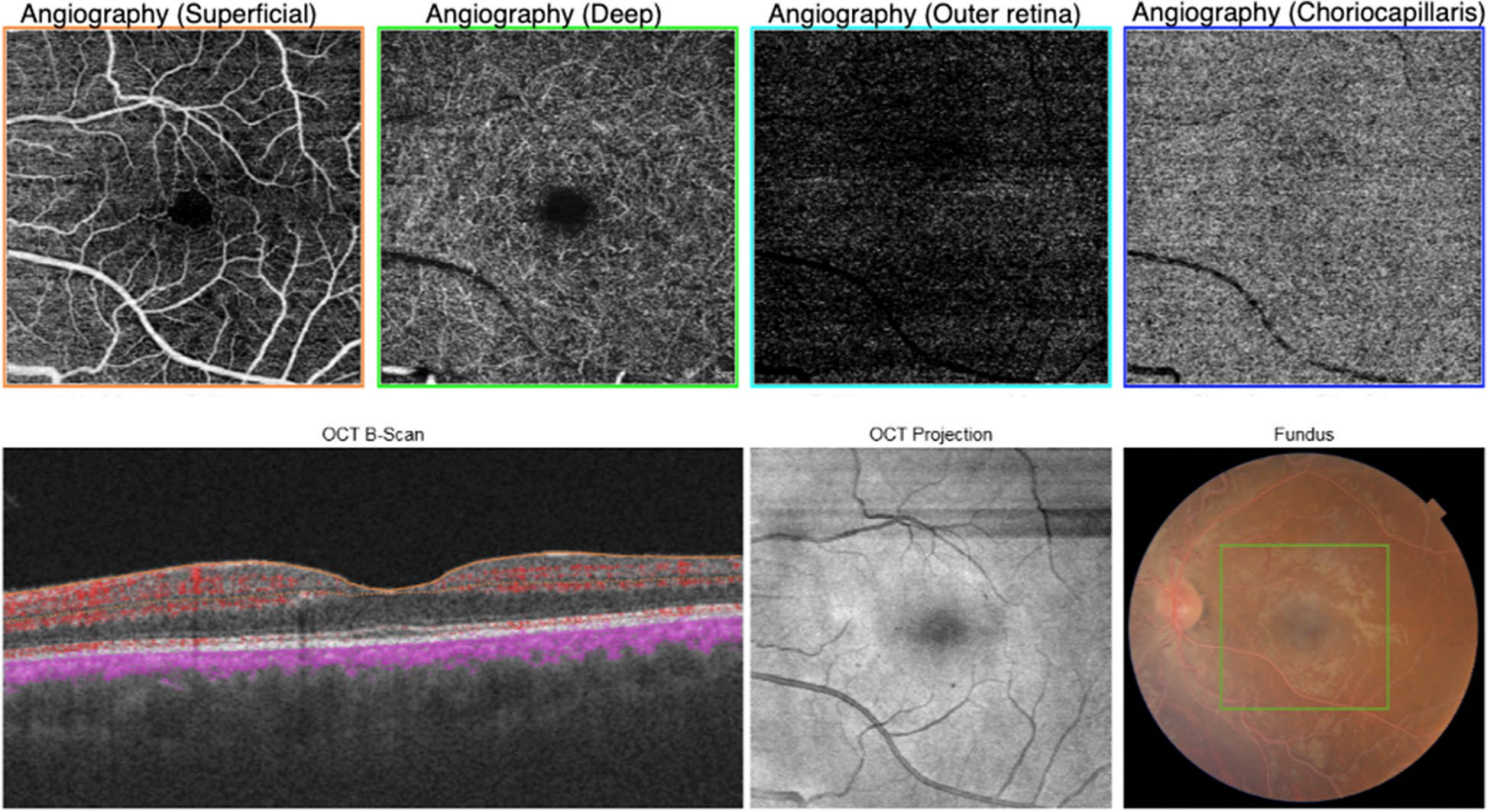
Swept-source optical coherence tomography (OCT)-angiography is observed to be unremarkable in a patient with untreated lipemia retinalis

This group comprised four eyes with a fundus examination showing a creamy discoloration of the blood vessels consistent with lipemia retinalis (Fig. 1) (Table 1). Visual acuity was 20/20 in both eyes with normal intraocular pressure. SD-OCT demonstrated the presence of hyperreflective dots within the inner retina (Fig. 2a). On OCT-angiography, the superficial retinal plexus, deep retinal plexus, outer retina, and choriocapillaris were intact in all eyes with lipemia retinalis (Fig. 3).

**Table 1:** Baseline features of patients with history of lipemia retinalis (10 eyes)

Serial number	Subgroup	VA	IOP	Fundus appearance	Triglyceride level (normal 30—150 mg/dL)
1	Untreated	20/20	15	Abnormal	4096.50
2	Untreated	20/20	14	Abnormal	4096.50
3	Untreated	20/20	17	Abnormal	2503
4	Untreated	20/20	15	Abnormal	2503
5	Resolved	20/20	16	Normal	160.32
6	Resolved	20/20	15	Normal	160.32
7	Resolved	20/20	14	Normal	535.90
8	Resolved	20/20	14	Normal	535.90
9	Resolved	20/20	13	Normal	713.90
10	Resolved	20/20	17	Normal	713.90

*VA* Visual acuity, *IOP* Intraocular pressure

### Resolved lipemia retinalis group

Visual acuity and fundus examination were normal in all eyes. Moreover, SD-OCT of all eyes demonstrated the presence of hyperreflective dots in the inner retina (Fig. 2b), while OCT-angiography was unremarkable.

### Retinal oximetry and laser speckle flowgraphy findings

A significant difference was observed in retinal vascular oxygen saturation and diameter between all groups (Table 2). Patients with untreated lipemia retinalis had a significantly higher retinal arteriolar and venular oxygen saturation (*p* < 0.001). Furthermore, patients with untreated lipemia retinalis had significantly smaller retinal arteriolar and venular diameters (*p* < 0.001). On LSFG, there was a significant difference in the overall and vessel MBR of the optic nerve between the three groups (*p* = 0.007 and 0.011, respectively). Patients with lipemia retinalis (resolved and untreated) exhibited a high overall and vessel MBR of the optic nerve compared to the control group (Table 3). Optic nerve and choroidal BOS were significantly elevated, and the resistivity index was significantly lower in the untreated and resolved cohorts (Table 3). There was no statistically significant difference in the choroidal MBRs (overall, vessel, and tissue) between the groups. Interocular correlation for all parameters is demonstrated in Table 4.

**Table 2:** Comparisons between controls and lipemia retinalis retinal oximetry findings using ANOVA with post hoc

	Controls Mean ± SD (*n* = 20)	Untreated lipemia retinalis Mean ± SD (*p*-value) (*n* = 4)	Resolved lipemia retinalis Mean ± SD (*p*-value) (*n* = 6)	*p*-value
Arteriolar oxygen saturation (%)	92.1 ± 4.7	152.3 ± 15.9	91.1 ± 6.9	*P* < 0.001
		vs control (*P* < 0.001) vs resolved (*P* < 0.001)	vs control (*P* = 0.766) vs untreated (*P* < 0.001)	
Venular oxygen saturation (%)	59.5 ± 5.9	120.5 ± 19.5	57.8 ± 7.7	*P* < 0.001
		vs control (*P* < 0.001) vs resolved (*P* < 0.001)	vs control (*P* = 0.676) vs untreated (*P* < 0.001)	
Arteriolar diameter (pixels)	12.1 ± 1.1	9.8 ± 1.3	13.3 ± 1.2	*P* < 0.001
		vs control (*P* = 0.001) vs resolved (*P* < 0.001)	vs control (*P* = 0.032) vs untreated (*P* < 0.001)	
Venular diameter (pixels)	15.9 ± 1.2	13.3 ± 1.9	15.1 ± 1.8	*P* = 0.008
		vs control (*P* = 0.002) vs resolved (*P* = 0.055)	vs control (*P* = 0.248) vs untreated (*P* = 0.055)

**Table 3:** Comparisons between controls and lipemia retinalis mean blur rate (MBR)

**Optic** **nerve** **MBRs**						
	Controls Mean ± SD (n = 20)	Untreated lipemia retinalis Mean ± SD (n = 4) (*p*-value)		Resolved lipemia retinalis Mean ± SD (*n* = 6)		*p*-value
MBR (overall)	21.5 ± 4.2	28.6 ± 7.3		29.1 ± 8.3		*P* = 0.007
		vs control (*P* = 0.027)	vs resolved (*P* = 0.892)	vs control (*P* = 0.007)	vs untreated (*P* = 0.892)	
MBR (vessel)	38.2 ± 8.1	50.2 ± 9.7		48.1 ± 9.3		*P* = 0.011
		vs control (*P* = 0.016)	vs resolved (*P* = 0.704)	vs control (*P* = 0.019)	vs untreated (*P* = 0.704)	
MBR (tissue)	12.0 ± 2.5	13.7 ± 4.1		15.0 ± 2.6		*P* = 0.070
		vs control (*P* = 0.284)	vs resolved (*P* = 0.456)	vs control (*P* = 0.027)	vs untreated (*P* = 0.456)	
BOS	74.0 ± 4.2	79.7 ± 7.3		82.1 ± 4.7		*P* = 0.002
		vs control (*P* = 0.038)	vs resolved (*P* = 0.433)	vs control (*P* = 0.001)	vs untreated (*P* = 0.433)	
RI	0.4 ± 0.05	0.31 ± 0.1		0.28 ± 0.07		*P* = 0.001
		vs control (*P* = 0.015)	vs resolved (*P* = 0.528)	vs control (*P* = 0.001)	vs untreated (*P* = 0.528)	
**Choroidal MBRs**						
	Controls Mean ± SD (*n* = 20)	Untreated lipemia retinalis Mean ± SD (*n* = 4)		Resolved lipemia retinalis Mean ± SD (*n* = 6)		*p*-value
MBR (overall)	6.4 ± 2.2	7.4 ± 2.0		7.9 ± 1.5		*P* = 0.260
		vs control (*P* = 0.379)	vs resolved (*P* = 0.710)	vs control (*P* = 0.127)	vs untreated (*P* = 0.710)	
MBR (vessel)	10.0 ± 4.4	11.6 ± 4.3		12.6 ± 2.6		*P* = 0.371
		vs control (*P* = 0.485)	vs resolved (*P* = 0.706)	vs control (*P* = 0.185)	vs untreated (*P* = 0.706)	
MBR (tissue)	5.2 ± 2.1	5.5 ± 1.9		6.1 ± 0.8		*P* = 0.599
		vs control (*P* = 0.797)	vs resolved (*P* = 0.611)	vs control (*P* = 0.797)	vs untreated (*P* = 0.611)	
BOS	68.3 ± 5.8	78.1 ± 4.3		78.0 ± 5.5		*P* < 0.001
		vs control (*P* = 0.003)	vs resolved (*P* = 0.973)	vs control (*P* = 0.001)	vs untreated (*P* = 0.973)	
RI	0.45 ± 0.06	0.33 ± 0.06		0.33 ± 0.08		*P* = 0.002
		vs control (*P* = 0.003)	vs resolved (*P* = 0.985)	vs control (*P* = 0.001)	vs untreated (*P* = 0.985)	

*MBR* Mean blur rate, *BOS* Blow out score, *RI* Resistivity index

**Table 4:** Interocular correlation of the optic nerve and choroidal mean blur rates (MBRs) and retinal oximetry parameters

Group	Parameter	Mean difference (RE minus LE)	*p*-value
**Optic nerve MBRs**			
Untreated	MBR (overall)	0.25	0.851
	MBR (vessel)	2.45	0.395
	MBR (tissue)	-0.75	0126
	BOS	9.85	0.412
	RI	-0.13	0.417
Resolved	MBR (overall)	-3.80	0.334
	MBR (vessel)	-0.50	0.931
	MBR (tissue)	0.57	0.722
	BOS	-6.00	0.131
	RI	0.87	0.119
Control	MBR (overall)	-0.91	0.552
	MBR (vessel)	-3.74	0.219
	MBR (tissue)	-0.66	0.372
	BOS	-0.69	0.498
	RI	0.01	0.457
**Choroidal MBRs**			
Untreated	MBR (overall)	-0.45	0.323
	MBR (vessel)	-1.65	0.519
	MBR (tissue)	-1.85	0.245
	BOS	-1.35	0.727
	RI	0.03	0.605
Resolved	MBR (overall)	0.59	0.616
	MBR (vessel)	0.68	0.566
	MBR (tissue)	0.86	0.480
	BOS	-6.66	0.022
	RI	7.56	0.017
Control	MBR (overall)	0.30	0.434
	MBR (vessel)	1.15	0.310
	MBR (tissue)	0.00	1.00
	BOS	-0.55	0.642
	RI	0.01	0.603
Retinal oximetry			
Untreated	Arteriolar oxygen saturation (%)	15.88	0.229
	Venular oxygen saturation (%)	-9.67	0.295
	Arteriolar diameter (pixels)	-0.20	-.097
	Venular diameter (pixels)	-0.57	0.660
Resolved	Arteriolar oxygen saturation (%)	0.35	0.763
	Venular oxygen saturation (%)	1.57	0.257
	Arteriolar diameter (pixels)	1.79	0.216
	Venular diameter (pixels)	-0.69	0.561
Control	Arteriolar oxygen saturation (%)	1.67	0.296
	Venular oxygen saturation (%)	3.31	0.123
	Arteriolar diameter (pixels)	-0.15	0.377
	Venular diameter (pixels)	-0.33	0.492

*RE* Right eye, *LE* Left eye, *MBR* Mean blur rate, *BOS* Blow out score, *RI* Resistivity index

## Discussion

The present study was conducted to assess the ocular blood flow and oxygen metabolism in patients with lipemia retinalis. Retinal oximetry illustrated high oxygen saturation within the retinal arterioles and venules. Since oxygen delivery is an outcome of blood flow, raised blood flow may increase oxygen saturation [22]. This may be responsible for protecting the photoreceptors and can elucidate the preservation of visual acuity and retinal function despite the severe vascular structural changes. In a study on central retinal vein occlusion and retinal oximetry, a negative correlation was observed between the retinal ischemic index and venular oxygen saturation [23]. The high venular oxygen saturation observed in lipemia retinalis may elucidate why this condition does not generally cause retinal ischemia. Patients with treated hypertriglyceridemia and normal fundi demonstrated lower levels of retinal oxygen saturation. These reversible retinal oximetry changes may provide a deeper understanding of the underlying physiological changes and can be used to monitor patients with lipemia retinalis. The reversibility of retinal oxygen saturation with treatment has also been reported in patients with Vogt-Koyanagi-Harada disease (VKH) [24, 25]. Results of oxygen saturation in untreated lipemia retinalis may be affected by some parameters. As Oxymap T1 retinal oximeter utilizes the difference in light absorption between oxygenated and deoxygenated hemoglobin in estimating oxygen saturation [22]. Creamy discoloration of retinal blood vessels may alter retinal vascular wavelengths and interfere with the results. Variability in results may be observed with narrower blood vessels diameters, however a cut off is usually set, and the machine typically excludes vessels below 56 um [22].

LSFG measures blood flow by detecting changes in contrast patterns in light scatter owing to red blood cell movement. In the present study, LSFG revealed an increase in retinal blood velocity. The high retinal blood flow is an important prognostic factor and may elucidate why photoreceptors are preserved, and subsequently, visual acuity remains unaffected in lipemia retinalis. The retinal blood flow is reduced in conditions with photoreceptor dysfunction as in retinitis pigmentosa and rhegmatogenous retinal detachment [26, 27]. Moreover, retinal blood velocity predicted the vision improvement in patients with central retinal vein occlusion who were receiving intravitreal anti-vascular endothelial growth factor (VEGF) injections [28, 29]. The preservation of vision in lipemia retinalis may also be owing to the unaffected choroidal MBRs in different disease stages. Choroidal MBRs or blood velocity was demonstrated to be low in the acute stage of different choroidal inflammatory disorders such as acute posterior multifocal placoid pigment maculopathy, punctate inner choroidopathy, and VKH [30–32]. An increased BOS, the blood flow persisting between heartbeats, and a low RI suggests that retinal vascular resistance is raised in patients with lipemia retinalis. A high BOS and low RI, observed in both the untreated and resolved groups, may suggest that vascular resistance may not be reversible. However, patients in the group with resolved lipemia retinalis did not reach the normal range of triglycerides. Hence, whether the increased vascular resistance may resolve when triglycerides are lowered to normal levels is unclear. Alternatively, triglycerides may affect retinal and choroidal BOS and RI regardless of the presence or absence of lipemia retinalis. Similarly, in a study conducted on glycosylated hemoglobin (HbA1c) and LSFG, the former was observed to affect the optic nerve and choroidal BOS, regardless of the presence of diabetes mellitus [33]. The decrease in vascular diameters observed in our study may be elucidated by high oxygen saturation since high extracellular oxygen may lead to vasoconstriction [34]. LSFG demonstrates that the choroidal blood vessel velocity is not affected, indicating that lipemia retinalis is primarily a retinal disease with minimal effects on choroidal vasculature.

OCT-angiography confirmed the retinal vessel patency at the superficial and deep retinal capillary plexuses and choriocapillaris, while SD-OCT revealed the presence of inner retinal hyperreflective dots. The latter on OCT is owing to the extravasated lipid deposition. Özturk et al. were the first to report the hyperreflective dots [15]. These changes were observed in the present study in patients with normal fundi and hence could be considered as a subclinical disease marker. The utilization of OCT-angiography in assessing the retinal perfusion and retinal vascular density (VD) was widely described in systemic diseases. It is found that the retinal perfusion and retinal VD are decreased in patients with systemic hypertension, stroke, and chronic kidney [35–37]. Nonetheless, the assessment of retinal vasculature using OCT-angiography was previously described in one patient with lipemia retinalis, which demonstrated intact superficial and deep retinal capillary plexuses [38]. These findings are consistent with our study.

Lipemia retinalis fundus changes appear typically when serum triglyceride levels exceed 2500 mg/dL [13]. However a recent report showed one case with a serum triglyceride level more than 2500 mg/dL and a normal fundus appearance and another case with a serum triglyceride level less than 2500 mg/dL and an abnormal fundus appearance [39]. This may suggest that other factors contributing to the abnormal fundus appearance in lipemia retinalis may exist. Grouping the patients based on triglyceride level and fundus appearance and not addressing other factors may be a limitation of the present study. Due to the disease scarcity, the study may be limited owing to the low number of patients with lipemia retinalis. Nevertheless, to the best of our knowledge, the present study clarifies the ocular microcirculation aspects of lipemia retinalis for the first time. Further multi-center studies on a larger number of patients may be needed to support our study findings.

## Conclusion

Lipemia retinalis is observed to be associated with significant alteration in ocular blood flow and retinal oxygenation saturation. The increase in retinal blood flow, as well as the unaffected choroidal blood flow, may elucidate the preservation of visual acuity and function despite the apparent vascular changes observed in lipemia retinalis. The high retinal blood flow may consider the high retinal oxygen saturation values observed in lipemia retinalis.

## Data Availability

The datasets generated and/or analysed during the current study are not publicly available due to privacy/ethical restrictions but are available from the corresponding author on reasonable request.
